# An Expanded Genetic Code Enables Trimethylamine Metabolism in Human Gut Bacteria

**DOI:** 10.1128/mSystems.00413-20

**Published:** 2020-10-27

**Authors:** Veronika Kivenson, Stephen J. Giovannoni

**Affiliations:** a Department of Microbiology, Oregon State University, Corvallis, Oregon, USA; Dalhousie University

**Keywords:** cardiovascular disease, microbiome, molecular genetics

## Abstract

Cardiovascular disease (CVD) has been linked to animal-based diets, which are a major source of trimethylamine (TMA), a precursor of the proatherogenic compound trimethylamine-*N*-oxide (TMAO). Human gut bacteria in the genus *Bilophila* have genomic signatures for genetic code expansion that could enable them to metabolize both TMA and its precursors without production of TMAO. We uncovered evidence that the *Bilophila* demethylation pathway is actively transcribed in gut microbiomes and that animal-based diets cause *Bilophila* to rapidly increase in abundance. CVD occurrence and *Bilophila* abundance in humans were significantly negatively correlated. These data lead us to propose that *Bilophila*, which is commonly regarded as a pathobiont, may play a role in mitigating cardiovascular disease. Human gut microbiomes have been shown to affect the development of a myriad of disease states, but mechanistic connections between diet, health, and microbiota have been challenging to establish. The hypothesis that *Bilophila* reduces cardiovascular disease by circumventing TMAO production offers a clearly defined mechanism with a potential human health impact, but investigations of *Bilophila* cell biology and ecology will be needed to fully evaluate these ideas.

**IMPORTANCE** Links between trimethylamine-*N*-oxide (TMAO) and cardiovascular disease (CVD) have focused attention on mechanisms by which animal-based diets have negative health consequences. In a meta-analysis of data from foundational gut microbiome studies, we found evidence that specialized bacteria have and express a metabolic pathway that circumvents TMAO production and is often misannotated because it relies on genetic code expansion. This naturally occurring mechanism for TMAO attenuation is negatively correlated with CVD. Ultimately, these findings point to new avenues of research that could increase microbiome-informed understanding of human health and hint at potential biomedical applications in which specialized bacteria are used to curtail CVD development.

## Abstract (precis)

Links between trimethylamine-*N*-oxide (TMAO) and cardiovascular disease (CVD) have focused attention on mechanisms by which animal-based diets have negative health consequences. In a meta-analysis of data from foundational gut microbiome studies, we found evidence that specialized bacteria have and express a metabolic pathway that circumvents TMAO production and is often misannotated because it relies on genetic code expansion. This naturally occurring mechanism for TMAO attenuation is negatively correlated with CVD. Ultimately, these findings point to new avenues of research that could increase microbiome-informed understanding of human health and hint at potential biomedical applications in which specialized bacteria are used to curtail CVD development.

## INTRODUCTION

The human gut microbiome is increasingly recognized for its influential role in health. The gut microbiome has been implicated in cardiovascular disease (CVD), the leading cause of death in developed countries (1). Metabolic end products associated with the consumption of animal products have been shown to promote CVD; dietary sources of proatherogenic compounds include choline, phosphatidylcholine, and l-carnitine (2–11). Gut microorganisms convert these compounds to trimethylamine (TMA), which in turn is converted to trimethylamine-*N*-oxide (TMAO) through the action of host hepatic flavin monooxygenase 3 (12, 13). TMAO is a causative agent of CVD pathogenesis; therefore, elucidating pathways relevant to this compound is central to understanding human health (10, 14).

**FIG 1:**
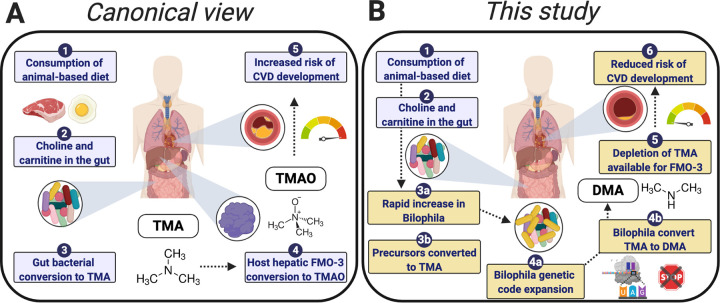
(A) Canonical view of TMA: this compound is converted to TMAO in the liver via a host hepatic flavin monooxygenase 3, leading to increased risk of cardiovascular disease. (B) A new view of TMA metabolism proposed in this study: the specialized gut bacteria, *Bilophila*, increase in abundance and use genetic code expansion to augment metabolism, thereby reducing the amount of TMA available for conversion to TMAO. TMA, trimethylamine; FMO-3, flavin monooxygenase 3; TMAO, trimethylamine-*N*-oxide; CVD, cardiovascular disease; DMA, dimethylamine.

The canonical view of CVD and TMAO involves the action of the gut microbiota in transforming precursor compounds from a range of animal-based dietary sources to TMA, which can then be converted to TMAO in the liver (Fig. 1A). While TMAO is frequently referred to as the sole breakdown product of TMA, a subset of methanogenic archaea in the gut have the ability to utilize TMA by an alternative pathway (15, 16). This pathway is enabled by genetic code expansion (GCE), via insertion of the 22nd amino acid, pyrrolysine (Pyl), in place of a TAG amber codon at conserved sites of the tri-, di-, and monomethylamine methyltransferase genes, with methane as the end product (17–19). TMA metabolism that relies on proteins that use an expanded code has also been described in some bacteria from environmental settings, including symbionts of gutless marine worms (20), as well as the *Firmicutes* bacterium Acetohalobium arabaticum isolated from a Crimean lagoon (21). Despite its potential importance, bacterial metabolism of TMA remains largely unexplored in the gut microbiome.

A Deltaproteobacterium commonly found in the human gut, Bilophila wadsworthia, also has genes necessary for encoding pyrrolysine (22). First identified in an appendicitis infection in 1989, this “bile-loving,” taurine-degrading bacterium is commonly referred to as a pathobiont associated with abscesses (23, 24). Additionally, *Bilophila* is able to produce hydrogen sulfide, and these bacteria may be linked to inflammatory bowel disease, colorectal cancer, and systematic inflammation (24–28). Despite its classification as a pathobiont, *Bilophila* is also commonly present in healthy human microbiomes (29, 30). There is uncertainty about GCE and TMA metabolic pathway characteristics in these organisms; notably, whether the pyrrolysine pathway is functional in *trans* and whether the pathway is expressed. The function of this pathway is questioned (21) because of organizational differences in the pyrrolysyl-tRNA synthetase gene (required for encoding Pyl): in archaea, this synthetase is encoded by a single gene, while in *Bilophila*, the N- and C-terminal domains are encoded by two distinct genes. Second, in previous reports of Pyl pathways, the trimethylamine methyltransferase and Pyl genes are commonly adjacent to one another (21, 22), while this is not the case in *Bilophila*. Third, the TMA methyltransferase protein is prematurely truncated at the TAG codon in public *Bilophila* data sets available on the NCBI and JGI-IMG webservers. Despite the ubiquity of this bacterium, and its potential importance in modulating TMAO levels (and thus CVD risk), these ambiguities remain unresolved, and studies of the human gut microbiome make no mention of the possibility of a bacterial pathway for TMA utilization. In this study, we investigate this alternative pathway of trimethylamine metabolism in *Bilophila*, the change in abundance of this taxon in response to an animal-based diet, and the correlation between *Bilophila* abundance and CVD pathology (Fig. 1B).

## RESULTS

**FIG 2:**
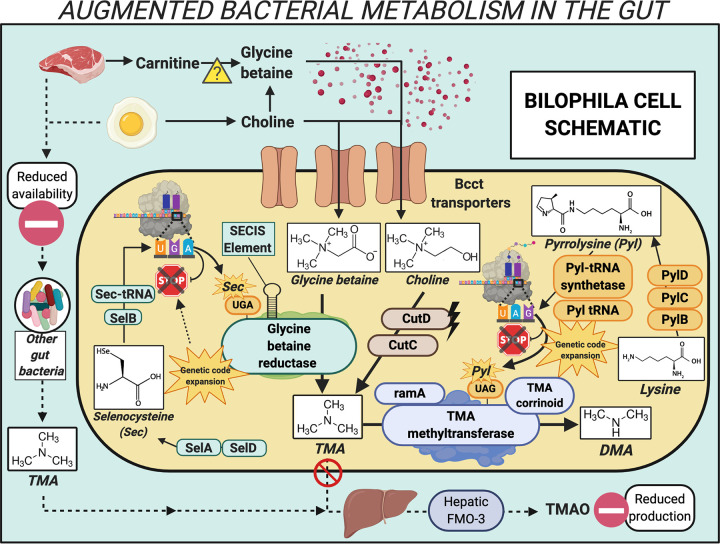
Mechanistic overview of genetic code expansion augmented metabolism performed by *Bilophila* in the human gut. Bcct transporters, betaine/carnitine/choline transporter family proteins; CutD, choline trimethylamine lyase activating enzyme; CutC, choline trimethylamine lyase; TMA, trimethylamine; DMA, dimethylamine; PylB, 3-methylornithine synthase; PylC, 3-methylornithine-l-lysine ligase; PylD, 3-methylornithyl-*N*^6^-l-lysine dehydrogenase; Pyl-tRNA synthetase, pyrrolysyl-tRNA synthetase; Pyl tRNA, pyrrolysine tRNA; ramA, methylamine methyltransferase corrinoid protein reductive activase; TMA corrinoid, methyltransferase cognate corrinoid protein; SelD, selenide, water dikinase; SelA, l-seryl-tRNA(Sec) selenium transferase; SelB, selenocysteine-specific translation elongation factor; Sec-tRNA, selenocysteine tRNA; SECIS Element, selenocysteine insertion sequence element; TMAO, trimethylamine-*N*-oxide; FMO-3, flavin monooxygenase 3.

To explore the potential for *Bilophila* TMA metabolism, we retranslated the protein-coding sequences from publicly available *Bilophila* genomes (Table S1) using a custom translation table with the TAG codon as a readthrough rather than a stop codon (see Materials and Methods). We found an in-frame TAG codon at a conserved position in the TMA methyltransferase, corresponding to the pyrrolysine residue of this protein in archaeal methanogens (17) and bacteria (20, 21) (Fig. 2 and Table S2). All genes known to have functions specific to pyrrolysine production were also identified in the *Bilophila* genomes: PylB, PylC, and PylD and the pyrrolysyl-tRNA synthetase N and C termini, as well as the Pyl-specific tRNA, with the corresponding CUA anticodon (Fig. 2; Fig. S1). One exception was that Bilophila wadsworthia ATCC 49260 is missing one component: a Pyl tRNA was not located. In addition to the Pyl-containing TMA methyltransferase, accessory genes for the TMA methyltransferase pathway, including *ramA* (the activating gene) and the TMA methyltransferase corrinoid protein, are encoded in all of the *Bilophila* genomes (Fig. 2 and Table S3). The dimethylamine methyltransferase gene is not encoded in any of the genomes, and a gene similar to monomethylamine methyltransferase, while present, does not have the conserved in-frame TAG codon that is a hallmark of this gene. In summary, metabolic inference from genomic data indicates that *Bilophila* have the potential to convert TMA to dimethylamine (DMA) in the human gut.

10.1128/mSystems.00413-20.1 FIG S1 The secondary structures of two tRNAs, for selenocysteine and pyrrolysine, from two *Bilophila* genomes. Structure of the pyrrolysine tRNA with CTA (CUA) anticodon (left) and selenocysteine tRNA with TCA (UCA) anticodon and extra arm (right) for (A) *Bilophila* sp. 4_1_30 and (B) Bilophila wadsworthia 3_1_6. Download FIG S1, PDF file, 0.2 MB. Copyright © 2020 Kivenson and Giovannoni. 2020 Kivenson and Giovannoni This content is distributed under the terms of the Creative Commons Attribution 4.0 International license.

10.1128/mSystems.00413-20.3 TABLE S1 Accession numbers for Bioprojects, corresponding publications, and reference numbers for *Bilophila* genomes used in this study. Download Table S1, PDF file, 0.04 MB. Copyright © 2020 Kivenson and Giovannoni. 2020 Kivenson and Giovannoni This content is distributed under the terms of the Creative Commons Attribution 4.0 International license.

10.1128/mSystems.00413-20.4 TABLE S2 Amino acid sequences of TMA methyltransferase proteins from *Bilophila* genomes, with the pyrrolysine residue (O) highlighted in green. Download Table S2, PDF file, 0.04 MB. Copyright © 2020 Kivenson and Giovannoni. 2020 Kivenson and Giovannoni This content is distributed under the terms of the Creative Commons Attribution 4.0 International license.

10.1128/mSystems.00413-20.5 TABLE S3 TIGRFAM identity and gene names of proteins relevant for *Bilophila* genetic code expansion and TMA metabolism. Download Table S3, PDF file, 0.04 MB. Copyright © 2020 Kivenson and Giovannoni. 2020 Kivenson and Giovannoni This content is distributed under the terms of the Creative Commons Attribution 4.0 International license.

The TMA-utilizing pathway we observed may benefit *Bilophila* by enabling them to use TMA directly and also TMA produced metabolically within the cell from two precursor compounds, choline and glycine betaine (Fig. 2). Briefly, *Bilophila* genomes encode the glycyl radical choline-TMA lyase and its associated activating protein (CutC and CutD, respectively), which convert choline to TMA (31). Glycine betaine (sources of which includes choline [32] and possibly carnitine [33]) can be converted to TMA via the selenocysteine (Sec)-containing glycine betaine reductase (GRD) pathway (34). The GRD pathway and the full set of machinery required for the noncanonical amino acid selenocysteine, encoded with a repurposed UGA stop codon, are also present in the *Bilophila* genomes (Table S3). Multiple copies of proteins belonging to the betaine/carnitine/choline family of transporters are also encoded in the *Bilophila* genomes. We conclude that the genomic data indicate that *Bilophila* has the ability to use choline and glycine betaine, converting these compounds to TMA, and subsequently to DMA. In doing so, these bacteria may deplete precursor compounds and TMA that would otherwise be available for host hepatic processes, thereby reducing or circumventing production of TMAO (Fig. 2).

Next, we asked whether the pyrrolysine pathway is functional and whether GCE-enabled TMA metabolism is active in *Bilophila* from the gut environment. To do so, we reexamined data sets from a recent human gut metatranscriptomic study (35) and a mouse model system study (36) (Table S1). Applying a minimum threshold of 98% amino acid sequence identity with annotated *Bilophila* proteins, we identified expression of the TMA methyltransferase and pyrrolysine machinery proteins in both human fecal and mouse cecum samples (Fig. 2 and Tables S4 and S5). The fraction of TMA consumed via this bacterial metabolic process in the human gut microbiome remains uncertain, but expression data support the conclusion that this metabolic process is active.

10.1128/mSystems.00413-20.6 TABLE S4 The metatranscriptomic data sets from mouse and human studies, the SRA accession numbers, and the number of reads for each data set. Download Table S4, PDF file, 0.06 MB. Copyright © 2020 Kivenson and Giovannoni. 2020 Kivenson and Giovannoni This content is distributed under the terms of the Creative Commons Attribution 4.0 International license.

10.1128/mSystems.00413-20.7 TABLE S5 The TMA and Pyl genes from metatranscriptomic data, their respective amino acid sequences, and the percent identity with corresponding proteins in the *Bilophila* genomes. Download Table S5, PDF file, 0.05 MB. Copyright © 2020 Kivenson and Giovannoni. 2020 Kivenson and Giovannoni This content is distributed under the terms of the Creative Commons Attribution 4.0 International license.

## DISCUSSION

This survey of published microbiome sequence data uncovered evidence that bacteria in the genus *Bilophila* use genetic code expansion in the human gut to produce a TMA methyltransferase. We hypothesized that this mechanism could be used to compete with other TMA-utilizing processes, potentially decreasing the production of TMAO from the proatherogenic precursor trimethylamine. To explore this hypothesis, we reexamined additional publicly available data (Table S1) to determine whether this naturally occurring mechanism for TMAO attenuation is correlated with CVD. In a recent study describing the gut microbiome in atherosclerotic cardiovascular disease, Jie et al. (29) report that *Bilophila* is one of the 20 most abundant genera in the samples examined for this project. Their data also show that the abundance of *Bilophila* is significantly enriched in the microbiomes of individuals in the healthy/control group (*n* = 187) compared to the CVD group (*n* = 218). Second, in a study describing a rapid diet-induced change in the human gut microbiome, David et al. (37) reveal that *Bilophila* significantly increase in abundance in response to an animal-based diet compared to a plant-based diet. Finally, in a study detailing the transmission of atherosclerosis susceptibility via gut microbial transplanation, Gregory et al. (38) show that mice with certain taxa have increased TMAO levels and develop atherosclerotic lesions and postulate that this is a microbiome-dependent, transmissible trait. *Bilophila* is one of only six taxa that are significantly enriched in both the cecal and fecal microbiome of the healthy group compared to the mice that developed atherosclerotic lesions. The observations we report may challenge the widely held idea that members of this taxon act exclusively as pathobionts; their role in the microbiome and human health may be context dependent, and their potential to mitigate the impacts of animal products on CVD warrants further study.

## MATERIALS AND METHODS

### Microbiome data selection and accession numbers.

Genomic, metagenomic, and metatranscriptomic data sets used for this study were accessed on 15 January 2020 from publicly available sequencing projects (see Table S1 in the supplemental material). The *Bilophila* genomes analyzed are reference genomes from the Human Microbiome Project (39) as well as the type strain for this genus, from the Refseq database (40). All of the available genomes from the genus *Bilophila* were examined. BioProject accession no. PRJEB33885 was excluded because it includes a duplicate of a previously published genome (included in accession no. PRJNA41963). Paired metagenomic-metatranscriptomic data were accessed from a large cohort study of adult men (35), and additional metatranscriptomic data were used from a mouse model system (36). Metatranscriptomic and metagenomic read data were obtained using the SRA-toolkit (https://github.com/ncbi/sra-tools) v2.9.1 data via the fastq-dump option.

### Genomic analysis and implementation of a custom protein translation table.

Predicted proteins in each genome were initially identified using Prodigal (42) v.2.6.3 using single genome mode, and functional annotation was determined using Hmmer (43) v.3.1, with the hmmscan option (1E−10 cutoff), with top hits only, against the Pfam (44) database v.31 and Tigrfam (45) database v.15.0.

For genomes in which enzymes were identified for the synthesis of pyrrolysine, we applied an alternate protein prediction procedure that reassigned TAG codons from stop to readthrough. To accomplish this task, we modified the source code for Prodigal by adding a custom translation table that has TAG readthrough and retains all three canonical bacterial start codons. Proteins with in-frame stop codons in the relevant genes were then manually inspected to determine whether the region containing and following the stop codon was conserved in comparisons to homologues containing pyrrolysine at a similar position (see Fig. S2 in the supplemental material). The modified code and documentation for the modified translation table are freely available at https://github.com/VeronikaKivenson/Prodigal. Protein searches for the full-length TMA methyltransferase amino acid sequence from *Bilophila* were performed on the NCBI and JGI-IMG webservers on 1 July 2020. For searching and identifying putative selenocysteine-containing proteins, the bSECIS (46) webserver was used and results were inspected and compared with previously identified selenoproteins. Protein sequence alignment was performed using Muscle (47), with Geneious 2020.1 used for visualization of the alignments. The Aragorn (48) web server was used to locate the tRNA sequences from each genome and to determine the secondary structure of Sec- and Pyl-specific tRNAs, as well as their corresponding anticodon sequences. tRNAscan-SE (49) v.1.23 was also used to search for the tRNAs for Sec. Chemdraw and Biorender software were used to create figures.

10.1128/mSystems.00413-20.2 FIG S2 Alignment of amino acid sequences from trimethylamine methyltransferase following the repurposed stop for *Bilophila* and an archaeon (Methanosarcina barkeri) that have pyrrolysine biosynthesis proteins; the sequence following the repurposed codon (pyrrolysine residue O) is conserved. Download FIG S2, PDF file, 0.07 MB. Copyright © 2020 Kivenson and Giovannoni. 2020 Kivenson and Giovannoni This content is distributed under the terms of the Creative Commons Attribution 4.0 International license.

### Metatranscriptomic data analysis.

Preliminary mapping of metatranscriptomic sequence data to *Bilophila* genomes was done using Bowtie2 (50) v2.3.4.1. This approach identified samples in which these *Bilophila* bacteria were present and active at detectable levels. Next, metatranscriptomic reads were coassembled using SRA data from select samples belonging to each BioProject (Table S4) using Megahit (51) v.1.1.1, with default parameters. Functional annotation and identification of stop codon readthrough were performed as described earlier. In addition to Prodigal, FragGeneScan was used to identify partial genes (52). The computational biology data processing and analysis workflow were completed using the Extreme Science and Engineering Discovery Environment (XSEDE) Bridges resource at the Pittsburgh Supercomputing Center (53, 54).
