# Supplementary material for: An Expanded Genetic Code Enables Trimethylamine Metabolism in Human Gut Bacteria
Source: mSystems. 2020 Oct 27;5(5):e00413-20. doi: 10.1128/mSystems.00413-20 (PMC7593587; doi:10.1128/mSystems.00413-20)
Supplement: FIG S1 [file mSystems.00413-20-sf001.pdf]

**A**

Bilophila sp. 4_1_30

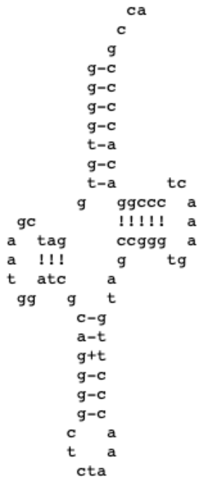

```
tRNA-Pyl(cta)
72 bases, %GC = 62.5
Sequence [437,508]
```

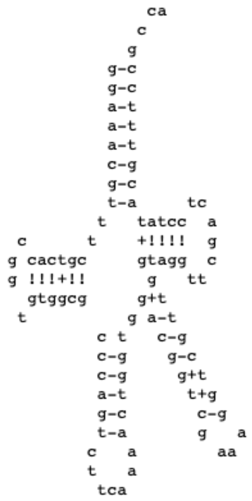

```
tRNA-SeC(tca)
94 bases, %GC = 55.3
Sequence c[862704,862797]
```

```
Primary sequence for tRNA-Pyl(cta)
1   .   10   .   20   .   30   .   40   .   50
ggggtgtggatcgaatggatcgcaggggctctaaacccttgtagccgggt
gaaactcccggacaccccgcca
```

```
Primary sequence for tRNA-SeC(tca)
1   .   10   .   20   .   30   .   40   .   50
ggaaacgtttcgtcaccggtgtggcgcccagtcttcaaaactggtggacg
gtcgaaaggtcgttggtaggttcgactcctatacgtttccgcca
```

**B**

Bilophila wadsworthia 3_1_6

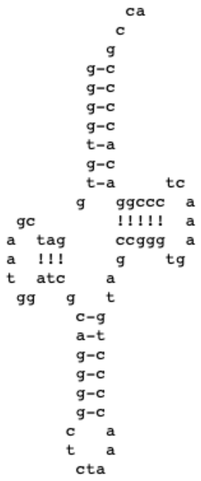

```
tRNA-Pyl(cta)
72 bases, %GC = 63.9
Sequence [735263,735334]
```

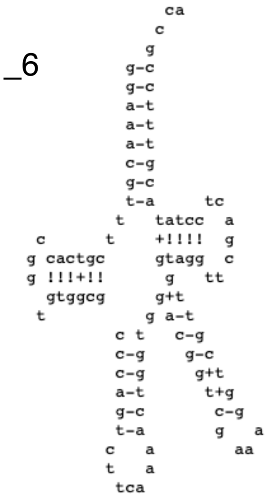

```
tRNA-SeC(tca)
94 bases, %GC = 55.3
Sequence c[70717,70810]
```

```
Primary sequence for tRNA-Pyl(cta)
1   .   10   .   20   .   30   .   40   .   50
ggggtgtggatcgaatggatcgcaggggctctaaaccccctgtagccgggt
gaaactcccggacaccccgcca
```

```
Primary sequence for tRNA-SeC(tca)
1   .   10   .   20   .   30   .   40   .   50
ggaaacgtttcgtcaccggtgtggcgcccagtcttcaaaactggtggacg
gtcgaaaggtcgttggtaggttcgactcctatacgtttccgcca
```
