# Supplementary material for: An Expanded Genetic Code Enables Trimethylamine Metabolism in Human Gut Bacteria
Source: mSystems. 2020 Oct 27;5(5):e00413-20. doi: 10.1128/mSystems.00413-20 (PMC7593587; doi:10.1128/mSystems.00413-20)
Supplement: TABLE S1 [file mSystems.00413-20-st001.pdf]

BIOPROJECT	***DATA TYPE***	***PUBLICATION / PROJECT***	***REF***
PRJNA354235	Metatranscriptomic, Human fecal	**Nature Microbiology 2018** Metatranscriptome of human faecal microbial communities in a cohort of adult men	35
PRJNA445875	Metatranscriptomic, mouse cecal	**Nature Communications 2018** Bilophila wadsworthia aggravates high fat diet induced metabolic dysfunctions in mice	36
PRJNA202303	Metagenomic, human fecal	**Nature Letters 2014** Diet rapidly and reproducibly alters the human gut microbiome	37
PRJEB21528	Metagenomic, human fecal	**Nature Communication 2017** The gut microbiome in atherosclerotic cardiovascular disease	29
PRJNA41961	Genomic, fecal Bilophila sp. 4_1_30	**Science 2010** A catalog of reference genomes from the human microbiome (HMP reference genome)	39
PRJNA41963	Genomic, fecal Bilophila_ 3_1_6_V2	**Science 2010** A catalog of reference genomes from the human microbiome (HMP reference genome)	39
PRJNA223063	Genomic, abscess B. wadsworthia ATCC 49260	**Nucleic Acids Res 2014** RefSeq microbial genomes database: new representation and annotation strategy	40
