# Supplementary material for: An Expanded Genetic Code Enables Trimethylamine Metabolism in Human Gut Bacteria
Source: mSystems. 2020 Oct 27;5(5):e00413-20. doi: 10.1128/mSystems.00413-20 (PMC7593587; doi:10.1128/mSystems.00413-20)
Supplement: TABLE S2 [file mSystems.00413-20-st002.pdf]

ORGANISM ID	***PYL- TMA METHYLTRANSFERASE AMINO ACID SEQUENCE WITH PYRROLYSINE HIGHLIGHTED***
Bilophila_ sp_4_1_30,	*MKRWAHSSLMGGVGVGLNFLREKDCEKIHEASLEVLHDRGAYFDSETAREVLRDHGCWED<br>ADGCTHFPRTLVESALEAVPAEFVHRGRTPDDDIHMAQDQVYASNFGEGIFTHDLETGER<br>RSTVKQDAVDILRVVDSLDNIHIYNRAIGPQDVPSESASMHNAEVAFCYTSKPMHLVSGS<br>PFQTKKMIKMAEIAAGGKEELKRRPRTAFNHTTISPLRISHEACENAIIVAEAGLPNHIL<br>VMVQQGATSPISYAGSVAVHNADFLAFNTLMQCVNRGNPTLYGASACVMDMKKGLSLVAA<br>PEVFVLNAAMARMSKYYNIPSYIAGG**O**TDSICNDVQAGAERALNRLMTALAGASVLFGQG<br>MLETGLTFDIPTLLVDDEIIDYVLRMLAGFKVDATTLSTDLIKEVGPFGTYLAEMDTFEH<br>LGDLSTYNLMNRRNYDMWAASGKPDLYGQARERAKEILATHKQKNPLSPEQVKAIRDVLV<br>DAEGELGVADFWKGKEEKRFIDNDLY*<br><br>***(Pyl at AA 327 of 506)***
Bilophila_ 3_1_6_V2,	*MKRWAHSSLMGGVGVGLNFLREKDCEKIHEASLEVLHDRGAYFDSETAREVLRDHGCWED<br>ADGCTHFPRTLVESALEAVPAEFVHRGRTPDDDIHMAQDQVYASNFGEGIFTHDLETGER<br>RSTVKQDAVDILRVVDSLDNIHIYNRAIGPQDVPSESASMHNAEVAFCYTSKPMHLVSGS<br>PFQTKKMIKMAEIAAGGKEELKRRPRTAFNHTTISPLRISHEACENAMIVAEAGLPNHIL<br>VMVQQGATSPISYAGSVAVHNADFLAFNTLLQCVNRGNPTLYGASACVMDMKKGLSLVAA<br>PEVFVLNAAMARMSKYYNIPSYIAGG**O**TDSICNDVQAGAERALNRLMTALAGASVLFGQG<br>MLETGLTFDIPTLLVDDEIIDYVLRMLAGFKVDATTLSTDLIKEVGPFGTYLAEMNTFEH<br>LGDLSTYNLMNRRNYDMWAASGKPDLYGQARERAKEILATHKQKTPLSPEQVKAIRDVLV<br>DAEGELGVADFWKGKEEKRFIDNDLY*<br><br>***(Pyl at AA 327 of 506)***
Bilophila_ 3_1_6_V2,	*MAQRWKKAGSLTTGGMSLNVFTQDEMDAIHRATLEVLEHTGVLIGSPEARALLVEAGASV<br>IDEDIVRFPQWMVADAVSCAPETLLLAGRTPDRDVVLDSTRVMFTNFGEAVYLIDPDTGE<br>VRNTTKKDVEKLTRVVDALDVIPVCERMAGAQDYPEQVAELHNYEALLMNTTKHVFTGGG<br>NGKLTQYMIDMAKAAVGEENFEERCPVTFNTCPISPLKLTADVCEVIMTAARNGATVNVL<br>SMGMAGGSTPVNLAGALVVHNCEALAGLVLAQTTRRGAKFIYGSSSTAMDLRYGAAVVGT<br>PELAVLNAGVAAMARYYKLPSWAAGG**O**GDSKCGDAQSGHEKTLTGLLPMLAGANIIYGLG<br>MLEMGMTISYSQLLMDAEMAEMMLFSMDGIVVNDETLSVDVIKEVGPRSDFLAHMNTFEN<br>MYIQSKPKLIDRLTRDRWNEAGHLDMESRALIAAKELLATWEPEPLPEEACARVRAVLNA<br>AERDYGVPESLE*<br><br>***(Pyl at AA 327 of 493)***

ORGANISM ID	***PYL- TMA METHYLTRANSFERASE AMINO ACID SEQUENCE WITH PYRROLYSINE HIGHLIGHTED***
Bilophila_ ATCC_49260	*MKRWAHSSLMGGVGVGLNFLREKDCEKIHEASLEVLHDRGAYFDSETAREVLRDHGCWED<br>ADGCTHFPRTLVESALEAVPAEFVHRGRTPDDDIHMAQDQVYASNFGEGIFTHDLETGER<br>RSTVKQDAVDILRVVDSLDNIHIYNRAIGPQDVPSESASMHNAEVAFCYTSKPMHLVSGS<br>PFQTKKMIKMAEIAAGGKEELKKRPRTAFNHTTISPLRISHEACENAMIVAEAGLPNHIL<br>VMVQQGATSPISYAGSVAVHNADFLAFNTLLQCVNRGNPTLYGASACVMDMKKGLSLVAA<br>PEVFVLNAAMARMSKYYNIPSYIAGG**O**TDSICNDVQAGAERALNRLMTALAGASVLFGQG<br>MLETGLTFDIPTLLVDDEIIDYVLRMLAGFKVDATTLSTDLIKEVGPFGTYLAEMNTFEH<br>LGDLSTYNLMNRRNYDMWAASGKPDLYGQARERAKEILATHKQKNPLSPEQVKAIRDVLV<br>DAEGELGVADFWKGKEEKRFIDNDLY**<br><br>***(Pyl at AA 327 of 506)***
Bilophila_ ATCC_49260	*MAQRWKKAGSLTTGGMSLNVFTQDEMDAIHRATLEVLEHTGVLIGSPEARALLVEAGASV<br>IDEDIVRFPQWMVADAVSCAPETLLLAGRTPDRDVVLDSTRVMFTNFGEAVYLIDPDTGE<br>VRNTTKKDVEKLTRVVDALDVIPVCERMAGAQDYPEQVAELHNYEALLMNTTKHVFTGGG<br>NGKLTQYMIDMAKAAVGEENFEERCPVTFNTCPISPLKLTADVCEVIMTAARNGATVNVL<br>SMGMAGGSTPVNLAGALVVHNCEALAGLVLAQTTRRGAKFIYGSSSTAMDLRYGAAVVGT<br>PELAVLNAGVAAMARYYKLPSWAAGG**O**GDSKCGDAQSGHEKTLTGLLPMLAGANIIYGLG<br>MLEMGMTISYSQLLMDAEMAEMMLFSMDGIVVNDETLSVDVIKEVGPRSDFLAHMNTFEN<br>MYIQSKPKLIDRLTRDRWNEAGHLDMESRALIAAKELLATWEPEPLPEEACARVRAVLNA<br>AERDYGVPESLE**<br><br>***(Pyl at AA 327 of 493)***
Methanosarcina barkeri ASM97002v1<br><br>(Archaeal reference sequence)	*MAKNNAVAGFNALNGVELNLFTTDELKAIHYATMEVLMDPGIQVSDPEARQIFKENGCEV<br>NEKTNVVKIPEYLVRKALQLAPSRFVLWGRDKKFNTVQECGGKVHWTCFGTGVKVCKYQD<br>GKYVTVDSVEKDIADIAKLCDWAENIDYFSLPVSARDIAGQGAQDVHETLTPLANTAKHF<br>HHIDPVGENVEYYRDIVKAYYGGDEEEARKKPIFSMLLCPTSPLELSVNACQVIIKGARF<br>GIPVNVLSMAMSGGSSPVYLAGTLVTHNAEVLSGIVLAQLTVPGAKVWYGSSTTTFDLKK<br>GTAPVGSPELGLISAAVAKLAQFYGLPSYVAGS**O**SDAKVPDDQAGHEKTMTTLLPALAGA<br>NTIYGAGMLELGMTFSMEQLVIDNDIFSMVKKAMQGIPVSEETLAVESIQKVGIGNNFLA<br>LKQTRQLVDYPSNPMLLDRHMFGDWAAAGSKDLATVAHEKVEDVLKNHQVTPIDADIFKD<br>MQAIVDKADKAFRGM**<br><br>***(Pyl at AA 333 of 496)***
