# Supplementary material for: An Expanded Genetic Code Enables Trimethylamine Metabolism in Human Gut Bacteria
Source: mSystems. 2020 Oct 27;5(5):e00413-20. doi: 10.1128/mSystems.00413-20 (PMC7593587; doi:10.1128/mSystems.00413-20)
Supplement: TABLE S3 [file mSystems.00413-20-st003.pdf]

TIGRFAM ID	GENE ID	Role
TIGR02369	trimeth_pyl: trimethylamine:corrinoid methyltransferase	Contains Pyl
TIGR0427	Rama_corrin_act: methylamine methyltransferase corrinoid protein reductive activase	Part of TMA pathway
TIGR02370	pyl_corrinoid: methyltransferase cognate corrinoid proteins, Methanosarcina family	Part of TMA pathway
TIGR04395	cutC_activ_rSAM: choline TMA-lyase-activating enzyme	Pre-TMA pathway
TIGR04394	choline_CutC: choline trimethylamine-lyase	Pre-TMA pathway
TIGR00842	bcct: transporter, betaine/carnitine/choline transporter (BCCT) family	Pre-TMA pathway
TIGR03912	PylS_Nterm: pyrrolysine--tRNA ligase, N-terminal region	Needed for Pyl
TIGR02367	PylS_Cterm: pyrrolysine--tRNA ligase, C-terminal region	Needed for Pyl
TIGR03910	pyrrolys_PylB: pyrrolysine biosynthesis radical SAM protein	Needed for Pyl
TIGR03909	pyrrolys_PylC: pyrrolysine biosynthesis protein PylC	Needed for Pyl
TIGR03911	pyrrolys_PylD: pyrrolysine biosynthesis protein PylD	Needed for Pyl
TIGR01918	various_sel_PB: selenoprotein B, glycine/betaine/sarcosine/D-proline reductase family	Contains Sec
TIGR01917	gly_red_sel_B: glycine reductase, selenoprotein B	Contains Sec
TIGR00475	selB: selenocysteine-specific translation elongation factor	Needed for Sec
TIGR00474	selA: L-seryl-tRNA(Sec) selenium transferase	Needed for Sec
TIGR00476	selD: selenide, water dikinase	Needed for Sec
