# Supplementary material for: An Expanded Genetic Code Enables Trimethylamine Metabolism in Human Gut Bacteria
Source: mSystems. 2020 Oct 27;5(5):e00413-20. doi: 10.1128/mSystems.00413-20 (PMC7593587; doi:10.1128/mSystems.00413-20)
Supplement: TABLE S4 [file mSystems.00413-20-st004.pdf]

DATA TYPE	BIOPRPOEJCT	ACCESSION	NO. READS
Human gut metatranscriptomics	PRJNA354235	SRR6038200	53999202
Human gut metatranscriptomics	PRJNA354235	SRR6038205	10583862
Human gut metatranscriptomics	PRJNA354235	SRR6038250	32555998
Human gut metatranscriptomics	PRJNA354235	SRR6038264	11343188
Human gut metatranscriptomics	PRJNA354235	SRR6038349	2347600
Human gut metatranscriptomics	PRJNA354235	SRR6038429	26532234
Human gut metatranscriptomics	PRJNA354235	SRR6038472	53814756
Human gut metatranscriptomics	PRJNA354235	SRR6038359	56244250
Mouse cecal metatranscriptomics	PRJNA445875	SRR6904614	58278596
Mouse cecal metatranscriptomics	PRJNA445875	SRR6904615	62005814
Mouse cecal metatranscriptomics	PRJNA445875	SRR6904616	58553182
Mouse cecal metatranscriptomics	PRJNA445875	SRR6904617	57362426

HUMAN FECAL SAMPLES	METATRANSCRIPTOMIC AMINO ACID SEQUENCE	Bilophila 3_1_6 % ID	Bilophila _4_1_30 % ID	Bilophila_ ATCC_49260 % ID
TMA methyl-transferase	MQAGAERALNRLMTALAGASVLFGQGMLETGLTFDIPTLLVDDEIIDYV	100%	100%	100%
TMA methyl-transferase	FGEGIFTHDLETGERRSTVKQDAVDILRVVDSLDNIHIYNRAIGPQDVPSESASMHNAEVAFCYTSKPMHLVSGSPFQTKKMIKMAEIAAGGKEELKRRPRTAFNHTTISPLRISHEACENAMIVAEAGLPNHILVMVQQGATSPISYAGSVAVHNADFLAFNTLMQCVNRG	100%	100%	100%
PylC	EAELRAYIPEGFDAEGWVLESYCPGPSFSLEICGTPGNYRIFQVTDLLMDEAFD	98%	100%	100%
Pyl synthetase N-term	WRMTDEDSAVYGETSDFVDKNGMELASSALGPHPLDAAWGIMENWVGIGF	100%	100%	100%
PylD	AGHLGFEADVLPADVPGFQLAKSGGFDLFIWADDDTYLAENILTGTVGENGRATGRGFATALIRMAARKRLDKRALVLGAGPVGCAGAETLALAGYE	100%	100%	100%

MOUSE CECAL SAMPLES	METATRANSCRIPTOMIC AMINO ACID SEQUENCE	Bilophila 3_1_6 % ID	Bilophila _4_1_30 % ID	Bilophila_ ATCC_49260 % ID
TMA methyl-transferase	YVGEENFEERCPVTFNTCPISPLKLTADVCEVIMTAARNGATVNVLSMGMAGGSTPVNLAGALVVHNCEALAGLVLAQTTRRGAKFIYGSSSTAMDLRYGAAVVGTPELAVLNAGVAAMARYYKLPSWAAGG**O**GDSKCGDAQSGHEKTLTGLLPMLAGANIIYGLGMLEMGMTISYSQLLMDAEMAEMMLFSMDGIVVNDETLSVDVIKEVGPSDFLAHMNTFENMYIQSKPKLIDRLTRDRWNEAGHLDMESRALIAAKELLATWEPEPLPEEACARVRAVLNAAERDYGVPESLE	100%	100%	99%
TMA methyl-transferase	QCVNRGNPTLYGASACVMDMKKGLSLVAAPEVFVLNAAMARMSKYYNIPSYIAGGOTDSICNDVQAGAERALNRLMTALAGASVLFGQGMLETGLTFDIPTLLVDDEIIDYVLRMLAGFKVDATTLSTDLIKEVGPFGTYLAEMNTFEHLG	99%	99%	99%
Pyl synthetase C-term	AGIEGWRMTDEDSAVYGETSDFVDKNGMELASSALGPHPLDAAWGIMENWVGIGFGLERLTMAATGESTMAKTGRSLSYLHGIRLRI	100%	99%	100%
PylB	WYACYQETHNRDLFTRLRLEQDYDRRKRTRLEAAGCGLLAEDGLLTGVGESAEDLADSILDMAREPLDQVRAMSYVPHESTFPSTAGDTLEERRAHELLAIAAMRLVMEDRLIPASLDVDGLEGLAMRLKAGANVVTSIVPSGCGLAGVASKDLDIENQRRSVAAVVRQLGVLGLEPALPGEYRAWVEQRRRGEER	99%	99%	99%

**MOUSE CECAL SAMPLES**	**METATRANSCRIPTOMIC AMINO ACID SEQUENCE**	**Bilophila 3_1_6 % ID**	**Bilophila _4_1_30 % ID**	**Bilophila_ ATCC_49260 % ID**
PylC	GDRKFLEGGGRMRVTIVGGGLQGVELCWLARKAGWGTLLVDERPAPPALRLADVFAQCDVTKLGGSGVLTR	100%	98%	100%
PylC	REMEAALLNLAEMLRLHGLMDLEVIQAPEGMRVLEIDARFPSQTPTAVWLSTGVNLAEHLAACFFPYAPGSGLGAPRFARYEHVLCKDGGLHFLGEHIMGQFGPLEPVNGFCGADEALVGGSLL	99%	98%	100%
PylD	GENGRATGRGFATALIRMAARKRLDKRALVLGAGPVGCAGAETLALAGYEVFLCDMDGEKARAACGALSGCTPCTPDDLSGLPLFECLLDAAPTNDFFP	99%	98%	100%
Pyl synthetase N-term	PLPNLPLPLPTSSFPSTLKSEIAMSETPTTRPAPKQRTYRKNQFLFALIGKMKLWPSRKGILHGIRTMEIAGDH	100%	100%	100%
