# Supplementary material for: An Expanded Genetic Code Enables Trimethylamine Metabolism in Human Gut Bacteria
Source: mSystems. 2020 Oct 27;5(5):e00413-20. doi: 10.1128/mSystems.00413-20 (PMC7593587; doi:10.1128/mSystems.00413-20)
Supplement: TABLE S5 [file mSystems.00413-20-st005.pdf]

		% IDENTITY with:	% IDENTITY with:	% IDENTITY with:
**PROTEIN ID, Human fecal**	PROTEIN SEQUENCE	**Bilophila 3_1_6**	**Bilophila 4_1_30**	**Bilophila _ATCC_ 49260**
TMA methyltransferase	MQAGAERALNRLMTALAGASVLFGQGMLETGLTFDIPTLLVDDEIIDYV	100%	100%	100%
TMA methyltransferase	FGEGIFTHDLETGERRSTVKQDAVDILRVVDSLDNIHIYNRAIGPQDVPSESASMHNAEVAFCYTSKPMHLVSGSPFQTKKMIKMAEIAAGGKEELKRRPRTAFNHTTISPLRISHEACENAMIVAEAGLPNHILVMVQQGATSPISYAGSVAVHNADFLAFNTLMQCVNRG	100%	100%	100%
Pyl biosynthesis protein PylC	EAELRAYIPEGFDAEGWVLESYCPGPSFSLEICGTPGNYRIFQVTDLLMDEAFD	98%	100%	100%
Pyl tRNA synthetase N-term	WRMTDEDSAVYGETSDFVDKNGMELASSALGPHPLDAAWGIMENWVGIGF	100%	100%	100%
Pyl biosynthesis protein PylD	AGHLGFEADVLPADVPGFQLAKSGGFDLFIWADDDTYLAENILTGTVGENGRATGRGFATALIRMAARKRLDKRALVLGAGPVGCAGAETLALAGYE	100%	100%	100%
**PROTEIN ID, Mouse cecal**				
TMA methyltransferase	YVGEENFEERCPVTFNTCPISPLKLTADVCEVIMTAARNGATVNVLSMGMAGGSTPVNLAGALVVHNCEALAGLVLAQTTRRGAKFIYGSSSTAMDLRYGAAVVGTPELAVLNAGVAAMARYYKLPSWAAGGOGDSKCGDAQSGHEKTLTGLLPMLAGANIIYGLGMLEMGMTISYSQLLMDAEMAEMMLFSMDGIVVNDETLSVDVIKEVGPSDFLAHMNTFENMYIQSKPKLIDRLTRDRWNEAGHLDMESRALIAAKELLATWEPEPLPEEACARVRAVLNAAERDYGVPESLE	100%	100%	99%
TMA methyltransferase	QCVNRGNPTLYGASACVMDMKKGLSLVAAPEVFVLNAAMARMSKYYNIPSYIAGGOTDSICNDVQAGAERALNRLMTALAGASVLFGQGMLETGLTFDIPTLLVDDEIIDYVLRMLAGFKVDATTLSTDLIKEVGPFGTYLAEMNTFEHLG	99%	99%	99%

Pyl tRNA synthetase C-term	AGIEGWRMTDEDSAVYGETSDFVDKNGMELASSALGPHPLDAAWGIMENWVGIGFGLERLTMAATGESTMAKTGRSLSYLHGIRLRI	100%	99%	100%
Pyl biosynthesis protein PylB	WYACYQETHNRDLFTRLRLEQDYDRRKRTRLEAAGCGLLAEDGLLTGVGESAEDLADSILDMAREPLDQVRAMSYVPHESTFPSTAGDTLEERRAHELLAIAAMRLVMEDRLIPASLDVDGLEGLAMRLKAGANVVTSIVPSGCGLAGVASKDLDIENQRRSVAAVVRQLGVLGLEPALPGEYRAWVEQRRRGEER	99%	99%	99%
Pyl biosynthesis protein PylC	GDRKFLEGGGRMRVTIVGGGLQGVELCWLARKAGWGTLLVDERPAPPALRLADVFAQCDVTKLGGSGVLTR	100%	98%	100%
Pyl biosynthesis protein PylC	REMEAALLNLAEMLRLHGLMDLEVIQAPEGMRVLEIDARFPSQTPTAVWLSTGVNLAEHLAACFFPYAPGSGLGAPRFARYEHVLCKDGGLHFLGEHIMGQFGPLEPVNGFCGADEALVGGSLL	99%	98%	100%
Pyl biosynthesis protein PylD	GENGRATGRGFATALIRMAARKRLDKRALVLGAGPVGCAGAETLALAGYEVFLCDMDGEKARAACGALSGCTPCTPDDLSGLPLFECLLDAAPTNDFFP	99%	98%	100%
Pyl tRNA synthetase N-term	PLPNLPLPLPTSSFPSTLKSEIAMSETPTTRPAPKQRTYRKNQFLFALIGKMKLWPSRKGILHGIRTMEIAGDH	100%	100%	100%
