# Supplementary material for: An Expanded Genetic Code Enables Trimethylamine Metabolism in Human Gut Bacteria
Source: mSystems. 2020 Oct 27;5(5):e00413-20. doi: 10.1128/mSystems.00413-20 (PMC7593587; doi:10.1128/mSystems.00413-20)
Supplement: FIG S2 [file mSystems.00413-20-sf002.pdf]

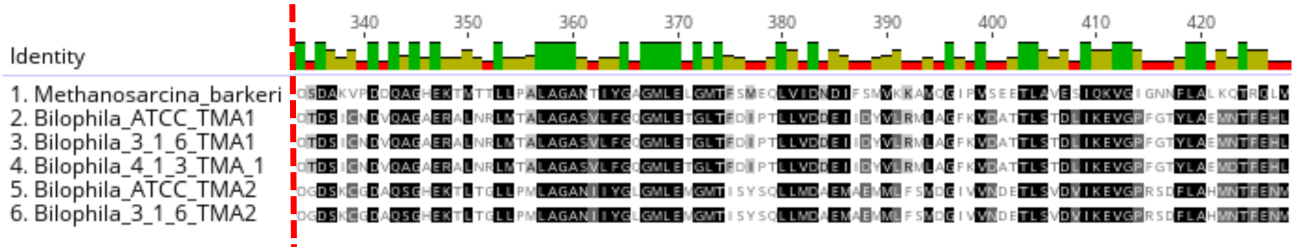
Identity
340
350
360
370
380
390
400
410
420
1. Methanosarcina_barkeri
2. Bilophila_ATCC_TMA1
3. Bilophila_3_1_6_TMA1
4. Bilophila_4_1_3_TMA_1
5. Bilophila_ATCC_TMA2
6. Bilophila_3_1_6_TMA2
